# Plasma Nitric Oxide and Acute Phase Proteins after Moderate and Prolonged xercises

**Published:** 2012

**Authors:** Akinwande Kazeem, Akinosun Olubayo, Arinola Ganiyu

**Affiliations:** 1 *Department of Chemical Pathology and Immunology, Faculty of Basic Medical Sciences, Olabisi Onabanjo University, Ago-iwoye, Nigeria *; 2 *Department of Chemical Pathology and Immunology, University College Hospital, Ibadan, Nigeria *

**Keywords:** Cardiovascular diseases, Exercises, Health benefits, Inflammation

## Abstract

**Objective(s):**

The aim of this study was to evaluate plasma levels of nitric oxide (NO) and certain acute phase proteins (caeruloplasmin, transferrin, haptoglobin, C-reactive protein) in Nigerian subjects after short and prolonged exercises.

**Materials and Methods:**

A total of 57 subjects (34 males and 23 females) between ages of 19 and 45 years participated in this study and were divided into three groups: Group 1 (Prolonged exercise) consisted of footballers (10 males and 9 females) who played football for at least two hrs daily; Group II (Moderate exercise) consisted of individuals (14 males and 5 females) who played football for 30 min 3 times a week; Group III (10 males and 9 females) were sedentary workers, who rarely had any form of physical exercise. The plasma samples were assayed for NO, C-reactive protein (CRP), caeruloplasmin, haptoglobin, and transferrin using spectrophotometer and immunoplates. Statistical analysis was done using the student’s-t-test.

**Results:**

The result showed that there was a significant reduction in the level of NO in prolonged exercise (*P*<0.05) when compared with control subjects while the increase of NO in subjects with moderate exercise was not statistically significant when compared with control subjects. C-reactive protein was significantly increased (*P*< 0.01) while transferrin and haptoglobin were significantly reduced (*P*< 0.001 and *P*< 0.01 respectively) in subjects with prolonged exercise when compared with control subjects. In moderate exercise, haptoglobin was significantly reduced (*P*< 0.05) while the reduction in the levels of caeruloplasmin and transferrin was not statistically significant when compared with control subjects. The mean level of CRP was significantly raised in prolonged exercise compared with controls or moderate exercise while the level of caeruloplasmin was significantly reduced in prolonged exercise compared with controls or moderate exercise.

**Conclusion:**

Moderate exercises should be encouraged.

## Introduction

Moderate exercise was found to improve immunity, reduce the risk of infection (1), increase cardiovascular functional capacity (2), improve cognitive functioning (3) and reduce incidence of major depression (4). Conversely intense exercise and over training have been thought to decrease immunity and raise infection risk, especially when coupled with emotional stress (5). Arner *et al *(6) reported that the oxidizability of LDL was enhanced in exercise, which imposes an oxidative stress and serves as an important deterrent of cardiovascular disease. Moreover, our previous study has suggested that vigorous exercise should be performed with caution due to its influence on certain blood biochemical parameters (7). 

Nitric oxide (NO) is an important signalling molecule that maintains the health of the vascular wall and regulates vasomotor function (8). *However, *exercise has been shown to augment endothelial- NO -dependent vasodilatation in both large and small vessels (8). It* has *also* been demonstrated that* both physical fitness and acute exercise regulate nitric oxide formation in healthy humans. Jungersten *et al *(9) concluded that physical fitness and formation of NO at rest are positively linked to each other and a single session of exercise elicits an acute elevation of NO formation. It was also demonstrated that nitric oxide synthetase activity is increased in skeletal muscle after an acute exercise (10). Inhibition of nitric oxide synthesis reduces exercise-induced vasodilation in the human forearm, indicating that nitric oxide plays a role in exercise-induced vasodilation (11).

An acute-phase protein has been defined as one whose plasma concentration increases (positive acute-phase proteins) or decreases (negative acute-phase proteins) by at least 25 percent during inflammatory disorders (12). Conditions that commonly lead to substantial changes in the plasma concentrations of acute-phase proteins include infection, trauma, surgery and burns, among others (13). Moderate changes in acute phase proteins occur after strenuous exercise, heatstroke, and childbirth. Small changes occur after psychological stress and in several psychiatric illnesses (14).

NO contributes to exercise hyperaemia and exercise leads to increase in blood flow and raised body temperature (15). Moreover, increased core temperature is among the beneficial effects of acute phase response (16). There is therefore the possibility that some relationship may exist between acute phase proteins, NO and exercises.

Based on the above rationale, the specific aim of this study is to determine the plasma levels of NO and some acute phase proteins in Nigerians during moderate exercise and long exercises compared with the controls. This will add to the existing level of information on the exercise which is beneficial. 

## Materials and Methods

***Subjects***

A total of 57 subjects (34 males and 23 females) between ages of 19 and 45 years participated in this study and were divided into three groups: Group I (prolonged exercise) consisted of footballers (10 males and 9 females) who played football for at least two hrs daily; Group II (Moderate exercise) consisted of individuals (14 males and 5 females) who played football for 30 min 3 times a week; Group III (10 males and 9 females) were sedentary workers, who rarely had any form of physical exercise. Groups I and II were selected from a sport centre in . Group III (10 males and 9 females) were sedentary staff of Federal Medical Centre, . The age ranges (mean ± S.D) of Group 1, Group II and Group III are between 19-44 years (32.6± 10), 20-45 years (34.1±6.6) and 19-45 years (32.3±8.4) respectively while the body mass indices of Group 1, Group II and Group III are 18.5±1.3, 19.6±2.1 and 21.4±4.8 respectively. A close ended questionnaire was administered detailing their age, marital status, type and duration of exercises, alcohol consumption, drug and smoking habits, presence of any disease(s) in the past or at present, presence of any form of inflammation, dietary habits and medication. Informed consent was obtained from all subjects before the commencement of the study.

Subjects excluded from the study included those who refused to give their consent, pregnant women, using substance of abuse, smokers, alcohol consumers, those cases with history of hypertension, heart failure and other disorders that predispose to cardiovascular diseases, subjects with inflammatory disease or infection and subjects that are obese.

After each category of exercise, ten millilitre of peripheral venous blood sample was drawn from the ante-cubital vein of each participant, using a tourniquet and a sterile, pyrogen – free needles and syringes into a lithium anticoagulant containing bottle. Blood samples were collected immediately after the bout of exercise because certain biochemical parameters change due to rest. All collected samples were sent to the laboratory within one hour of collection where they were centrifuged at 3,500 rpm for 5 min after which the plasma was separated and stored-frozen at -20^ o^C until analysis.

***Determination of plasma nitric oxide concentration using the Greiss method (17)***

The Greiss Reagent system Cat. No G2930, manufactured by Promega Corporation, was used.

***Principle of assay***

The assay is based on a chemical reaction which uses sulphanilamide and N -1-napthylethylenediamine dihydrochloride (NED) under acidic (phosphoric acid) conditions. Nitrite forms a coloured chromophore with the reagent, with an absorbance maximum at 543 nm wavelength, which is measured with an enzyme-linked immunosorbent assay (ELISA) microplate reader. The production of nitrite was quantified by comparing the result with absorbances of standard solutions of sodium nitrite.

***Determination of acute phase proteins concentration using the single radial immunodiffusion method***

The acute phase proteins were quantified by the single radial immunodiffusion method using immunoplates as previously described (18). 

***Data analysis***

Data were presented as MeanStandard Deviation. Student t-test (using pooled variance) was used to test the significance of difference between the mean values. The probability (*p*) values less than 0.05 were considered significant. The statistical analyses were done using SPSS version 15.0.

## Results

Table 1 and 2 show a comparison of the mean (±SD) value of NO and acute phase proteins in the test subjects compared with controls. There was a statistically significant decrease in plasma NO level in the subjects after prolonged exercise while there was no significant difference in NO level after moderate exercise when compared with the controls. There was also a significant difference in NO levels after moderate and prolonged exercises (Table 1)

**Table 1:** Comparison of the mean (±SD) values of nitric oxide in short and prolonged exercise to that of control subjects

Subjects	N	Nitric oxide (μmol/l)
Controls	19	46.6 ± 6.1
Moderate exercise	19	48.3 ± 6.0
Prolonged exercise	19	43.7 ± 1.0
t-test, *P* value^a^	0.866, *P*> 0.20	
t-test, *P* value^b^	2.045, *P*< 0.05*	
t-test, *P *value^c^	2.362,* P*< 0.05*	

Index: a – Controls compared with moderate exercise; b – Controls compared with prolonged exercise; c – Short exercise compared with prolonged exercise; * - Significantly different

**Table 2:** Comparison of the mean (±SD) values of acute phase proteins in short and prolonged exercise to that of control subjects

SUBJECTS	N	C-reactive protein (mg/dl)	Caeruloplasmin (mg/dl)	Transferrin (mg/dl)	Haptoglobin (mg/dl)
Controls	19	28.3 ± 11.1	92.0 ± 16.0	129 ± 12	600 ± 41
Moderate exercise	19	30.0 ± 10.1	89.0 ± 13.0	126 ± 10	563 ± 54
Prolonged exercise	19	38.6 ±9.1	81.0 ± 15.0	101 ± 14	554 ± 49
t-test, *P*value^a^		0.494, *P*> 0.50	0.634, *P*> 0.50	0.837,*P*> 0.20	2.379, *P*< 0.05*
t-test, *P* value^b^		3.128, *P*< 0.01*	1.829, *P* > 0.05	6.619, *P* < 0.001*	3.138,* P*< 0.01*
t-test, *P* value^c^		2.757, *P*< 0.01*	1.757, *P* > 0.05	6.334, *P*< 0.001*	0.538, *P* > 0.50

Index: a – Controls compared with moderate exercise; b – Controls compared with prolonged exercise; c – Short exercise compared with prolonged exercise; * - Significant .

Table 2 shows a comparison of the mean (±SD) values of C-reactive protein, caeruloplasmin, transferrin and haptoglobin after moderate and prolong exercises with controls. There was either significantly increased level of C-reactive protein or significantly reduced level of transferrin and haptoglobin after prolonged exercise compared with the controls. There were also significant differences in the mean levels of C-reactive protein and transferrin between subjects after moderate and prolonged exercises.

## Discussion

This study determined the effect of moderate and prolonged exercises on the plasma levels of nitric oxide and some acute phase proteins. In the present study, NO was significantly reduced after prolonged exercise when compared with control subjects. Our result corroborates the reports of Jungersten *et al *(9) who demonstrated an increased level of plasma and urinary nitrate (a major stable end product of nitric oxide metabolism) in athletes and non – athletes after two hours of acute exercise, compared to basal level before exercise. Another study by Verges *et al *(19) also suggested that prolonged exercise induces a reduction in NO concentration within the lung that lasts for several minutes after the end of exercise.

The body undergoes some morphologic changes due to long exercise which includes increased muscle mass, dilated vessels as well as hypertrophy of the myocardium (athletic heart) and generation of oxidative damage (20). Thus, it may be conjectured that NO defence was used as an antioxidant during prolonged exercise. 

Several studies have demonstrated acute phase response in exercise. Strachan *et al *(21) showed a distant-related acute phase response in exercise as indicated by significantly raised serum CRP concentrations in long distance runners competing in events ranging from 15 to 88 km but only a small rise in CRP concentrations was seen after races of less than 21 km. This is in accordance with the result of present study which shows an increase in CRP levels after both moderate and prolonged exercises. 

CRP is a sensitive marker of systemic low-grade inflammation (22) and elevated plasma levels of CRP have been associated with an increased risk of coronary cardiovascular disease (23) in individuals who have no prior cardiovascular disease. Increased level of CRP after prolonged exercise might indicate acute phase response and inflammation.

Transferrin is an acute phase protein which increases following inflammation and transports iron, along with ferritin. Iron plays a key role in a number of cellular processes such as DNA synthesis and electron transport and it is an essential component of haemoglobin (24). A study demonstrated no change in transferrin (21) following long exercise. It has however shown that, during running, runners have significantly higher iron turnover and cell destruction level than other athletes (25). It could therefore be said that reduced transferrin level observed in this study might be due to several cell destruction and an increased iron turnover during prolonged exercise.

Haptoglobin is an acute phase protein, which specifically binds to haemoglobin and prevents external leak of iron (25). Strenuous exercise generally provides red cells with physical and chemical stresses, and promotes fragility of the red cell membrane and haemolysis (26). 

Reduced level of haptoglobin in prolonged exercise might be an indication of intravascular hemolysis during exercises.

## Conclusion

This study shows that, inflammation as well as production of oxidants takes place during prolonged exercise. The present study therefore corroborates a previous suggestion (7) that prolonged exercise should be undertaken with caution. 
